# Natural and Synthetic Anticancer Epidrugs Targeting the Epigenetic Integrator UHRF1

**DOI:** 10.3390/molecules28165997

**Published:** 2023-08-10

**Authors:** Waseem Ashraf, Tanveer Ahmad, Nicolas Reynoird, Ali Hamiche, Yves Mély, Christian Bronner, Marc Mousli

**Affiliations:** 1 Department of Pharmacology, Faculty of Pharmacy, Bahauddin Zakariya University, Multan 60800, Pakistan; waseem.ashraf@bzu.edu.pk; 2 Institut Pour L’avancée des Biosciences, Centre de Recherche UGA, INSERM U1209, CNRS 5309, Université Grenoble Alpes, 38058 Grenoble, France; tanveer.ahmad@univ-grenoble-alpes.fr (T.A.); nicolas.reynoird@univ-grenoble-alpes.fr (N.R.); 3 Department of Functional Genomics, Institut de Génétique et de Biologie Moléculaire et Cellulaire (IGBMC), INSERM U1258, CNRS UMR 7104, Université de Strasbourg, Equipe Labellisée Ligue Contre le Cancer, 67401 Illkirch, France; ali.hamiche@igbmc.fr; 4 Laboratoire de Bioimagerie et Pathologies, UMR 7021 CNRS, Faculté de Pharmacie, Université de Strasbourg, 67401 Illkirch, France; yves.mely@unistra.fr

**Keywords:** cancer, DNA methylation, epidrugs, epigenetics, phytochemical, tumor-suppressor genes UHRF1

## Abstract

Cancer is one of the leading causes of death worldwide, and its incidence and mortality are increasing each year. Improved therapeutic strategies against cancer have progressed, but remain insufficient to invert this trend. Along with several other risk factors, abnormal genetic and epigenetic regulations play a critical role in the initiation of cellular transformation, as well as tumorigenesis. The epigenetic regulator UHRF1 (ubiquitin-like, containing PHD and RING finger domains 1) is a multidomain protein with oncogenic abilities overexpressed in most cancers. Through the coordination of its multiple domains and other epigenetic key players, UHRF1 regulates DNA methylation and histone modifications. This well-coordinated dialogue leads to the silencing of tumor-suppressor genes (TSGs) and facilitates tumor cells’ resistance toward anticancer drugs, ultimately promoting apoptosis escape and uncontrolled proliferation. Several studies have shown that the downregulation of UHRF1 with natural compounds in tumor cells induces the reactivation of various TSGs, inhibits cell growth, and promotes apoptosis. In this review, we discuss the underlying mechanisms and the potential of various natural and synthetic compounds that can inhibit/minimize UHRF1’s oncogenic activities and/or its expression.

## 1. Introduction

Cancer is the second leading cause of death after cardiovascular pathologies. The incidence of cancer is increasing in both developing and developed countries, and approximately 10 million people died of this deadly disease in 2018 [1]. The increased prevalence of cancer is often correlated with hereditary traits, environmental pollution, bad lifestyles with poor dietary habits, and a lack of physical activity, leading to the oncogenic transformation of cells [2]. Epigenetic alterations play a pivotal role in these transformations, which are heritable changes in gene functioning without altering the DNA sequence [3]. These alterations tend to disrupt the overall balance between tumor-suppressor genes and oncogenes in favor of the latter, thus promoting cellular proliferation and transformation. Because of their reversible nature, scientists are actively investigating the aberrant epigenetic mechanisms in cancer to target them for therapies [4]. Key epigenetic mechanisms include DNA methylation, histone modifications, and non-coding RNA regulation [5,6]. Aberrant DNA methylation and histone modifications play a major role in tumorigenesis and the epigenetic mediators involved in these mechanisms have been well explored for anticancer targeting. So far, many drugs targeting DNA methylation (azacitidine and decitabine) and histone deacetylation (vorinostat, romidepsin, belinostat, Panobinostat, and chidamide) have been approved by regulatory bodies for chemotherapies [7,8,9]. Interestingly, the protein UHRF1 is one of the few proteins involved in both DNA methylation and histone modification regulation, thereby making it a prime target for anticancer therapy.

## 2. Structure and Function of UHRF1

### 2.1. Structure of UHRF1

**Figure 1:**
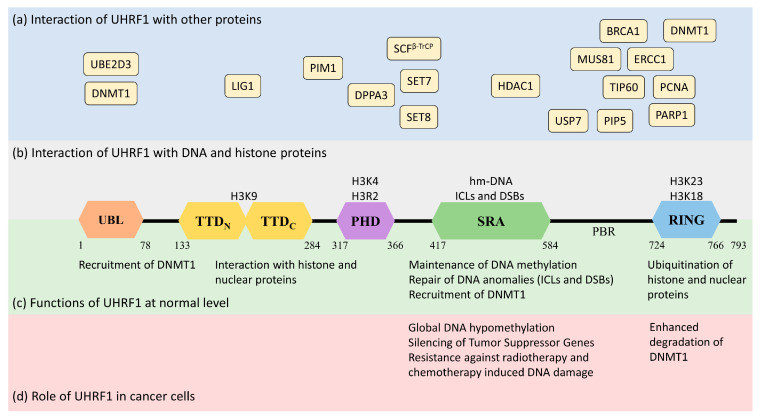
Structure and functions of UHRF1 protein (isoform 1) in normal and cancer cells along with its interaction with the DNA genome and nuclear proteins. UHRF1 is a multidomain protein having ubiquitin-like domain (UBL), tandem Tudor domain (TTD), plant homeodomain (PHD), SET and RING-associated domain (SRA), and really interesting new gene domain (RING) in its structure. (**a**) (in blue) indicates the interaction of different nuclear proteins with the proposed domains of UHRF1. (**b**) (in grey) indicates the interaction of UHRF1 with DNA and histone proteins. The SRA domain of UHRF1 recognizes hemi-methylated DNA or anomalies in DNA structure (ICLs and DSBs). TTD, PHD, and RING domains of UHRF1 interact with the indicated amino acids in H3 histone proteins. (**c**) (in green) highlights the cellular functions of UHRF1 in normal cells. UHRF1 is implicated in DNA methylation maintenance by recruiting DNMT1, recognizing DNA damage and initiating the DNA damage response, and regulating the function and stability of nuclear proteins through ubiquitination. (**d**) (in red) indicates the role of UHRF1 in cancer cells. UHRF1 represses many tumor-suppressor genes by maintaining the hyper-methylation of their promoters. High levels of UHRF1 in cancer cells also promote genetic instability by destabilizing DNMT1, which induces global hypomethylation. Furthermore, increased levels of UHRF1 facilitate the repair of DNA damage induced by radio or chemotherapy, making cancer cells resistant to anticancer therapy.

Human UHRF1 (initially known as ICBP90) was discovered by two of us as a transcriptional regulator of topoisomerase IIα by binding to an inverted CCAAT box in its promoter region [10,11]. However, it is now well-characterized for its involvement in various epigenetic and cellular pathways, including the maintenance of methylation patterns on DNA, histone modifications, DNA damage repair, and the regulation of other proteins [12,13,14]. UHRF1 is a 793-amino-acid-long protein coded by the *UHRF1* gene mapped at the 19p13.3 location in the human genome (Figure 1). UHRF1 is a protein with multiple domains that differ in their structure and functions [15,16]. Starting from the N-terminus, the domains are the ubiquitin-like domain (UBL), tandem Tudor domain (TTD), plant homeodomain (PHD), set and ring-associated (SRA) domain, and, finally, the really interesting new gene (RING) domain at the C-terminus of the protein (Figure 1).

It is interesting to note that UHRF1 shares a full sequence identity of 52% with UHRF2, another member of the UHRF family of proteins. The sequence identity is highest between the RING (79%) and the SRA (77%) domains of UHRF1 and UHRF2. However, despite their high sequence similarity, the expression profiles, the distributions, and the functions of these proteins are different [17].

#### 2.1.1. Ubiquitin-like Domain (UBL)

The UBL is structurally 35% identical to ubiquitin and is linked to the stability of UHRF1. It possesses characteristic α-helix and β-sheets, as well as two conserved lysine residues, K31 and K50 (similar to ubiquitin K29 and K48), which can be polyubiquitinated to trigger proteasomal degradation [12]. A role of the UBL has also been identified in DNMT1 (DNA methyltransferase 1) recruitment and the accurate transmission of methylation patterns [16]. Indeed, the UBL coordinates with the UHRF1 RING domain to ubiquitinate histone H3 residues, which serve as an anchorage signal for DNMT1 recruitment on hemi-methylated DNA [18,19]. Interestingly, the direct interaction of UHRF1-UBL with the RFTS (Replication Focus-Targeting Sequence) domain of DNMT1 also facilitates the enzymatic activity of the latter by activating its catalytic domain [20].

#### 2.1.2. Tandem Tudor Domain (TTD)

The Tandem Tudor domain of UHRF1 is involved in various protein–protein interactions vital for UHRF1 biological functions. The TTD notably allows UHRF1 to interact with the histone marks needed for its functioning [21]. The TTD is made up of two subdomains (TTDN and TTDC), each having a characteristic five-stranded β-barrel moiety in its structure [22]. The aromatic cage (Phe-152, Tyr-188, and Tyr-191) in TTDN, along with Asn-194 and Asp-145, recognizes di- and tri-methylated H3K9, while the peptide-binding groove between the TTDN and TTDC is sensitive to the epigenetic modifications on adjacent histone residues (i.e., H3K4 methylation and H3T6 phosphorylation) [22]. Through its interaction with LIG1 (DNA ligase), the TTD allows UHRF1 recruitment to replication foci to play its role in DNA methylation [23], but this interaction is not essential for abnormal DNA methylation patterns in cancerous cells [24]. The TTD also interacts with LIG1K126me3, which opens the closed conformation of UHRF1 [25,26]. In contrast, when the TTD interacts with the polybasic region (PBR) of UHRF1, the binding of H3K9me3 with the TTD-PHD domains is disrupted, ultimately returning to the closed conformation of UHRF1. Finally, when UHRF1 binds to hemi-methylated DNA or USP7 (Ubiquitin-Specific-processing Protease 7), the interaction of the TTD with the PBR is disturbed, which favors the transition to the open conformation of UHRF1 [27,28,29].

#### 2.1.3. Plant Homeodomain (PHD)

The plant homeodomain (PHD) of UHRF1 differs from the canonical PHDs in other proteins by having a PHD motif containing four cysteines (C302, C305, C313, and C316) in a loop coordinated with a zinc atom linked to the canonical PHD region by a single helical turn. The PHD domain of UHRF1 specifically recognizes the H3R2 motif in chromatin, which is essential for UHRF1 functions [30,31]. This interaction of the PHD with histone proteins can be altered through other proteins, such as DPPA3 (developmental pluripotency-associated protein 3), inhibiting UHRF1 localization on chromatin and promoting passive demethylation [32].

#### 2.1.4. Set and Ring-Associated (SRA) Domain

The SRA is a highly conserved domain specific to the UHRF family of proteins [12], playing an important role in DNA methylation [33,34,35]. Through this domain, UHRF1 recognizes hemi-methylated DNA and flips the methylated cytosine out of the helix. The SRA domain functions as a palm of a hand grasping the DNA duplex, where the NKR finger (489–491 amino acid residues) and thumb (444–496 amino acid residues) form two specific loops that project into major and minor grooves of the DNA double helix to read the nucleotides in the CpG duplex. The NKR finger specifically identifies the hemi-methylated DNA and flips the methylated cytosine out of the duplex. The flipped methylated cytosine is later stabilized by π-π stacking with the conserved tyrosine (466 and 478) residues in the binding domain [33]. The NKR domain also helps UHRF1 to differentiate between hemi-methylated and fully methylated DNA, since the second methylated cytosine creates a steric hindrance for the NKR finger, leading to a reduced affinity of UHRF1 for methylated DNA [33]. The only other protein from vertebrates carrying an SRA domain is UHRF2 [11]. In contrast to UHRF1, UHRF2 exhibits tumor-suppressor gene capacities [11,17], but an ability to favor tumor progression cannot be excluded, at least in certain types of cancer, such as hepatocellular carcinoma [36] or intestinal tumorigenesis [37]. The homology of amino-acid sequences between UHRF1 and UHRF2 reaches 74% [11,17], questioning the possibility that a drug targeting the SRA domain of UHRF1 putatively also may bind to the SRA domain of UHRF2 and thus may have opposite pharmacological effects. This seems, however, unlikely, but not impossible, considering the structures of each SRA domain. First, the UHRF2 SRA domain shows preferential recognition of hydroxymethyl cytosine over methylcytosine [38], suggesting differences in the respective structures. Indeed, the NKR loop, involved in the base-flipping mechanism, is disordered in the UHRF2-SRA domain, while in the UHRF1-SRA domain, it is not. This difference has been proposed to explain why the UHRF2-SRA domain has a preference for fully hydroxymethylated DNA over hemi-hydroxymethylated DNA vs. the UHRF1-SRA domain having a preference for hemi-methylated DNA over fully methylated DNA [38]. Altogether, this supports the fact that drugs targeting the SRA domains of UHRF1 would have specificity versus UHRF2, despite their strong similarities (77% amino acid sequence identity, personal observations).

#### 2.1.5. RING Domain

The RING domain harbors the only enzymatic activity of this protein [39]. It is rich in cysteine residues that form two zinc fingers interacting with a variety of substrates. Through this domain, UHRF1 can ubiquitinate itself, but also DNMT1, H3, and other proteins, regulating their function and stability [19,40].

### 2.2. Functions of UHRF1

**Figure 2:**
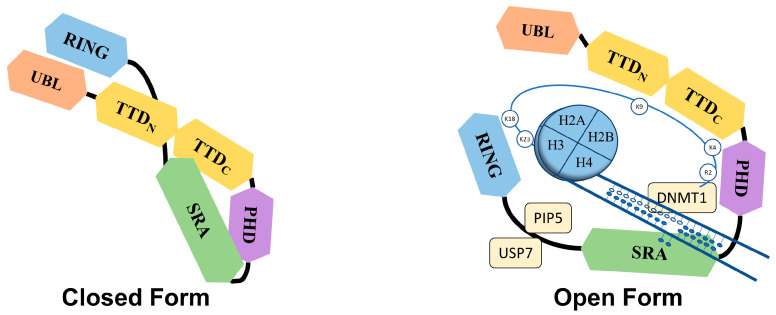
Proposed closed and open conformations of UHRF1. The PBR region between the SRA and RING domains interacts with TTD, which prevents the interaction of TTD with H3K9me3 marks, thus keeping the UHRF1 in closed conformation. On the other hand, proteins like PIP5 and USP7 can interact with the PBR of UHRF1 and disrupt the association between PBR and TTD, allowing TTD to interact with H3K9 methylation marks, which renders the UHRF1 in an open conformation. This open conformation of UHRF1 is also promoted by the binding of the SRA domain with hemi-methylated DNA, particularly in the S phase of the cell cycle.

UHRF1 is an important component of an epigenetic complex acting after DNA replication. UHRF1 is primarily involved in the maintenance of genomic DNA methylation patterns in cells [12,41]. Via its SRA domain, UHRF1 recognizes the CpG motifs on the parent strand of hemi-methylated DNA and flips the methylated cytosine out of the duplex. This then enables DNMT1 to methylate the opposite cytosine on the newly formed daughter strand [33,34,35,42]. UHRF1 also helps in recruiting DNMT1 to the hemi-methylation sites by direct interaction through its UBL and SRA domains, or indirectly by ubiquitinating histone H3K18 through its RING domain, as H3K18ub serves as a binding site for DNMT1. The crosstalk of UHRF1 TTD with H3K9me3 and H3K4 or PHD with H3R2 also plays a significant role in the recognition of the methylation site and the maintenance of the methylation patterns on the daughter strand [22,43,44,45]. The recruitment and activity of UHRF1 at the replication site is also facilitated by the DNA replication machinery. DNA ligase 1 (LIG1), methylated by G9a and GLP methyltransferases, mimics H3K9me2/3 in binding to the TTD of UHRF1. This binding recruits UHRF1 to the DNA replication sites for the maintenance of DNA methylation [23]. Similarly, the interaction with hemi-methylated DNA, USP7, and PIP5 (Phosphatidylinositol-4-phosphate 5) during the S-phase, triggers UHRF1 to switch from its “closed” to its “open” state, which facilitates UHRF1 loading onto the newly formed DNA [27,29,46] (Figure 2).

Along with its role in epigenetics, UHRF1 is also implicated in the DNA damage response and helps to maintain the integrity and stability of the genome. Initially, it was observed that the inhibition or depletion of UHRF1 leads to increased sensitivity to irradiation and higher accumulation of γH2AX in irradiated cells [47,48]. This function of UHRF1 in the DNA damage response is attributed to its SRA and RING domains, where the SRA domain acts as a sensor for DNA damage and the RING domain ubiquitinates the interacting proteins and directs the DNA repair pathways [47,48]. 

Owing to its SRA domain, UHRF1 identifies interstrand crosslinks (ICLs) and promotes DNA damage repair [49,50]. UHRF1 accumulation at damage sites precedes the recruitment of important effector proteins, such as FANCD2 (Fanconi anemia complementation group D2), which, in turn, is necessary for the recruitment of other factors of the Fanconi anemia (FA) repair pathway [50]. UHRF1, along with its paralogue UHRF2, forms a complex that monoubiquitinates FANCD2, which further enhances its retention after recruitment at the site of DNA damage [51]. UHRF1 also aids in the recruitment of ERCC1 (excision repair cross-complementation group 1) and MUS81 (crossover junction endonuclease MUS81 enzyme) at the site of ICLs through direct interactions with the SRA and RING domains, working as a nuclease scaffold to facilitate the ICL repair independently of the FA pathway [49]. It is very important to note that the recognition of ICLs by UHRF1 and facilitation in the DNA damage response is more pronounced in the S phase of the cell cycle and with ICLs showing minor distortions. 

UHRF1 is also associated with DNA damage repair resulting from double-strand breaks (DSBs), where it facilitates DNA repair by homologous recombination (HR) [52]. During DNA replication, UHRF1 accumulates at DSBs through its interaction with the BRCT (BRCA1 C-terminal) domain of BRCA1 (breast cancer gene 1) and phosphorylation of UHRF1 at Ser 674 by CDK2/cyclin. UHRF1, in turn, ubiquitinates the replication timing regulatory factor 1 (RIF1) at lysine K63, which results in a disruption of RIF1/53BP1 (p53-binding protein 1) foci formation, favoring DNA damage repair through the homologous recombination (HR) pathway by the accumulation of BRCA1 protein at the site of damage. Later, it was also reported that phosphorylated UHRF1 was prone to methylation by SET7 (histone-lysine N-methyltransferase) during the S phase, which enhanced the interaction between UHRF1 and PCNA (proliferating cell nuclear antigen) proteins, resulting in polyubiquitination of PCNA, thus stimulating DNA repair by the homologous recombination pathway [53]. This presence of DNA damage repair through the HR pathway is further augmented by the interaction of methylated UHRF1 with PARP1 [(poly(ADP-ribose) polymerase 1)], promoting cell cycle progression and increasing the DNA repair efficiency [54]. From all these studies, it can be concluded that UHRF1 undoubtedly plays an important role in DNA repair, but the intimate mechanism in which it is involved remains a mystery. Does it play a role in DNA sequence repair and/or the restoration of the DNA methylation patterns on the repaired DNA fragments? Further studies are required to fully decipher its role in DNA repair processes.

## 3. UHRF1 Expression and Its Regulation

UHRF1 is mostly associated with the proliferation potential of cells. Indeed, high levels of UHRF1 mRNA are found in proliferating tissues, such as the thymus, bone marrow, and liver, while there is marginal expression of UHRF1 in differentiated or quiescent cells [11]. The levels of UHRF1 also correlate with topoisomerase II expression, and UHRF1 is predicted as a cell-proliferation marker [11]. It was later observed that the UHRF1 levels peaked during the G1 and G2/M phases in normal cells, but were found to be consistently high in cancer cells [55]. High levels of UHRF1 facilitate the transition of cells to the S phase, while the downregulation of UHRF1 leads to the activation of the G1/S and G2/M checkpoints and cell cycle arrest [56,57]. The expression of UHRF1 is also regulated by several proteins, including E2F1 (E2F transcription factor 1), E2F8 (E2F transcription factor 8), FOXM1 (forkhead box protein M1), SP1 (specificity protein 1), SHMT2 (serine hydroxymethyltransferase-2), Setd1a (SET domain containing 1A, histone lysine methyltransferase), and Hmx1 (H6 family homeobox 1). Moreover, the activity of CD47 (cluster of differentiation 47) integrins increases UHRF1 expression in different cancers and represses the activity of many tumor-suppressor proteins [58,59,60,61,62,63]. Finally, the downregulation of various regulatory mi-RNAs leads to a high-level expression of UHRF1 proteins in cancers [64,65].

In addition, the activity and stability of UHRF1 can also be regulated by different post-translation modifications (PTMs). The SCFβ-TrCP enzyme can ubiquitinate UHRF1, leading to its degradation by the proteasomal pathway [66]. Deubiquitinase enzyme USP7 (known also as HAUSP) protects UHRF1 from ubiquitination-mediated degradation during the S phase, while during the M phase, USP7 dissociates from the phosphorylated UHRF1 [67]. Acetyltransferase TIP60 (also known as KAT5), which is an important epigenetic partner of UHRF1, regulates the stability of UHRF1 [68,69]. TIP60 interferes with the USP7–UHRF1 association and promotes the degradation of UHRF1 through auto-ubiquitination [68]. Through its intrinsic E3 ligase activity, UHRF1 can ubiquitinate itself (auto-ubiquitination) or other proteins, such as DNMT1, PAF15 (PCNA-associated factor 15), and histone proteins, and can regulate their function and stability [39,70]. Zhang et al. reported that TIP60 acetylates UHRF1 at K659 *in vitro*, which reduces its association with USP7 [29]. SET8 (SET domain-containing lysine methyltransferase 8, also known as SETD8 and KMT5A) regulates UHRF1 in the G2/M phase, though its methylation at K385 and ubiquitination at K500 leads to its degradation [71]. On the other hand, the demethylase LSD1 (lysine-specific demethylase 1) stabilizes UHRF1 through its demethylation activity and prevents its degradation [71]. UHRF1 interacts with HSP90 (heat shock protein 90), and the inhibition of HSP90 results in the ubiquitination of UHRF1, which is independent of its E3 ligase activity [72]. The phosphorylation of UHRF1 at serine 298 by protein kinase A favors its ability to induce the expression of topoisomerase IIα, which is critical for G1–S phase transition [73]. Protein kinase 2 (also known as CK2) can also phosphorylate UHRF1, enhancing its transcriptional activity, which is essential in G1–S phase transition and cellular proliferation [74]. On the other hand, the phosphorylation of UHRF1 at serine 311 by PIM1 (Proto-oncogene serine/threonine-protein kinase) can promote the degradation of UHRF1 and induce cellular senescence [75]. Casein kinase 1δ phosphorylates UHRF1 at serine 95 and degrades UHRF1 through SCF^β-TrCP^-mediated ubiquitination [66]. Altogether, these studies show that there are many putative regulatory hotspots that can lead to the dysregulation of UHRF1 expression. They also show that UHRF1 is a multi-target protein whose expression must be finely regulated to prevent tumorigenesis. These data also indicate that targeting UHRF1 may have strong therapeutic potential.

## 4. Why Targeting UHRF1 Is an Interesting Direction to Promote

The levels of UHRF1 have been found to be upregulated in many cancers [12,13,14,15,76]. Indeed, high levels of UHRF1 were observed in different types of cancers, including breast cancer, cervical cancer, colorectal cancer, prostate cancer, lung cancer, ovarian cancer, gall bladder cancer, retinoblastoma, osteosarcoma, and hepatocellular carcinoma [17,77]. The upregulation of UHRF1 is associated with high proliferation, invasion, and metastasis, notably by silencing many TSGs (tumor-suppressor genes), including *p14*, *p16^INK4A^*, *p21*, *p53*, *SLIT3* (slit guidance ligand 3), *CDH4* (cadherin 4), *RUNX3* (RUNX family transcription factor 3), *FOXO4* (forkhead box protein O4), *PPARG* (peroxisome proliferator-activated receptor gamma), *BRCA1* (breast cancer gene 1), *KLF17* (Krueppel-like factor 17), *PML* (promyelocytic leukemia), *CDH1* (cadherin 1), *PSP94* (prostatic secretory protein-94), *RARB* (retinoic acid receptor beta), *KISS1* (gene coding kisspeptin-54), *RGS2* (regulator of G-protein signaling 2), and *KEAP1* (Kelch-like ECH-associated protein 1) [64,77,78,79,80,81,82,83,84,85,86]. The silencing of TSGs is frequently associated with the hypermethylation of their promoters, resulting in reduced expression [87,88]. However, high levels of UHRF1 also induce the global hypomethylation of the genome by destabilizing DNMTs and promoting tumorigenesis as a result of chromosomal instability and the activation of imprinted genes, retrotransposons, and oncogenes [81,89,90] (Figure 1). Furthermore, UHRF1 upregulation in cancers also induces resistance to anticancer therapy [91,92,93]. High levels of UHRF1 were observed to confer resistance to radiotherapy in breast and esophageal cancer cells, associated with increased levels and activity of Ku70 (Lupus Ku autoantigen protein p70) and Ku80 (Lupus Ku autoantigen protein p80) DNA damage-repair proteins [48,94]. Recently, the UHRF1/BRCA1 DNA repair complex was shown to constitute an interesting target to treat cancer as it sensitizes the cells to DNA damage by giving them less opportunity to initiate DNA repair [95]. Therefore, all these studies highlight that UHRF1 is a highly attractive candidate for developing new anticancer drugs. Hereafter, we describe the current status of drug development aiming to target UHRF1 in a direct way or in a regulatory pathway context.

## 5. Synthetic Molecules Targeting UHRF1

Currently, much effort is being devoted to designing small synthetic molecules to antagonize the functions of UHRF1 [96,97,98]. The aim is to find more effective and safer molecules for treating cancers than the traditional chemotherapeutic agents endowed with high toxicities. In this section, we have summarized the ongoing research on small synthetic inhibitors of UHRF1 and their application as anticancer agents (Table 1).

### 5.1. Molecules Directly Targeting UHRF1 Protein

#### 5.1.1. Targeting the SRA Domain of UHRF1

The SRA domain of UHRF1 plays a crucial role in recognizing hemi-methylated (HM) DNA and recruiting DNMT1 to replication foci [34,99]. Thus, the SRA domain appears to be a promising target for screening molecules for anticancer therapy. Small molecules that bind to the SRA domain can perturb the binding of UHRF1 with HM DNA and thus regulate the methylation levels in cancers. A pioneering study used a tandem virtual screening approach to explore molecules that can impact the SRA/HM DNA complex [100]. The compound NSC232003 was identified, which binds to the SRA-binding pocket and regulates UHRF1 function by preventing the UHRF1/DNMT1 interaction [100]. This molecule is thought to fit into the 5-methyl cytosine (5-mC) binding pocket of the SRA domain of UHRF1 and disrupt the interaction between the UHRF1 SRA domain and HM DNA. It also impacts the interaction between UHRF1 and DNMT1, leading to global hypomethylation [100]. This investigation provided the first evidence of the druggability of the SRA domain to modulate DNA methylation.

Using fluorescence nucleobase analogs, our team designed a new approach to explore the detailed mechanism of how the SRA domain reads DNA and flips 5-mC [101]. This sensitive approach provided new insights into the UHRF1/DNMT1 interaction and was also used to screen small molecules that can inhibit base flipping [101]. By using this approach in combination with virtual screening, we sought UHRF1 inhibitors that could target the 5-mC binding pocket of the SRA domain and inhibit 5-mC base flipping. Out of 26 compounds selected by virtual screening, we were able to identify an anthraquinone compound named UM63 (2-amino-3-hydroxyanthra-9,10-quinone) that efficiently targeted the 5-mC binding pocket of the SRA domain and inhibited the flipping of 5-mC [98]. We also showed that UM63 prevented DNMT1 recruitment on the replication foci and reduced the methylation levels. This study shed light on the key role of base flipping in the recruitment of DNMT1 and confirmed the druggability of the 5-mC binding pocket of the SRA domain [98]. However, UM63 showed undesirable DNA intercalation properties, which motivated us to search for novel UHRF1-SRA inhibitors based on the UM63 structure using a multidisciplinary approach [102]. Two compounds, AMSA2 (a hydroxyanthracene derivative, namely anthrarobin) and MPB7 (an imidazoquinoline derivative) inhibitors, were identified. Both compounds inhibit the UHRF1-SRA-mediated base flipping at low micromolar concentrations without showing DNA intercalation properties [102]. UHRF1 base flipping inhibition further prevented DNMT1 recruitment at replication foci, leading to global hypomethylation [102]. The inhibitors also induced cell death in various cancer cell lines (HeLa: cervical cancer, A375: melanoma, and T47D: breast ductal carcinoma) while having a minimal effect only on non-cancerous cells (foreskin fibroblasts). This higher sensitivity of cancer cells over normal cells was attributed to the higher expression of UHRF1 in cancer cells [102]. Overall, both AMSA2 and MPB7 compounds appear as interesting leads for the development of new anticancer drugs.

Another screening approach based on time-resolved fluorescence resonance energy transfer (TR-FRET) was used to identify small molecules capable of preventing the interaction of SRA with HM DNA. Using this technique and a library called LOPAC (Library of Pharmacologically Active Compounds), seven compounds, including common anti-cancer compounds (mitoxantrone, idarubicin, doxorubicin, pixantrone, and daunorubicin) were validated as SRA inhibitors. These compounds induced global demethylation and exhibited a synergistic effect when combined with decitabine, a DNMT inhibitor [103].

#### 5.1.2. Targeting the TTD Domain of UHRF1

A small-molecule fragment screening identified a molecule named BPC (4-benzylpiperidine-1-carboximidamide) [104] that interacts with the TTD groove of the UHRF1 protein and favors the open conformation of UHRF1. In its open conformation, UHRF1 binds with low affinity to trimethylated histone H3 (H3K9me3), which alters UHRF1 functions. Therefore, the allosteric targeting of UHRF1 by small molecules can modulate the UHRF1-mediated histone code reading for experimental or therapeutic purposes. In 2018, using an H3K9me3 displacement assay and a library of 44,000 compounds, two compounds preventing TTD binding to H3K9me3 were identified [105]. These compounds, called NV01 and NV03, were, however, not sufficiently potent to enter cell-based assays, but could initiate the development of new anti-cancer drugs [105]. Moreover, *in silico* screening identified 5A-DMP (5-amino-2,4,-dimethylpyridine) as a novel ligand for the TTD [106]. The crystal structure of the TTD-5A-DMP complex showed that 5A-DMP could stably bond to the arginine-binding cavity of the TTD and inhibited its interaction with LIG1 [106].

### 5.2. Synthetic Drugs Affecting UHRF1 at the Transcriptional Level and/or Protein Level

Extracellular signal-regulated kinases 1/2 (ERK1/2) play an important role in cellular proliferation and are overexpressed in breast and liver cancers [107,108]. The activation of ERK1/2 leads to the release of the transcription factor E2F, which binds to the promoter region of UHRF1, ultimately regulating UHRF1 expression [109]. Inhibitors of ERK1/2 (LY294002, GF109203X, D98059, AG490, and genistein) inhibit cellular proliferation and colony formation in a dose-dependent mode in Jurkat cells [109]. The treatment of Jurkat cells (acute leukemic cells) showed a significant decline in the expression levels of phosphorylated ERK1/2 and UHRF1 proteins (especially PD98059, LY294002, and AG490) [109]. These data indicate that UHRF1 may be targeted by inhibiting the ERK1/2 signaling pathway [109]. Moreover, a recent study by Li et al. showed that the activated MEK/ERK pathway is responsible for the abnormal overexpression of UHRF1 and DNMT1. Indeed, the MEK1/2 inhibitor (PD0325901), in combination with the GSK3β inhibitor (CHIR99021), down-regulated both proteins at transcriptional levels [110].

Dihydroartemisinin (DHA), an artemisinin derivative, has anti-apoptotic and anti-proliferative activities in prostate cancer cells [111,112]. DHA treatment significantly reduces the mRNA levels of UHRF1 in a concentration-dependent manner. It down-regulates the UHRF1 and DNMT1 protein levels and up-regulates *p16^INK4A^* through the demethylation of its promoter. It also induces apoptosis and cell cycle arrest at the G1/S phase in prostate cancer cells, which indicates its potential as an effective anti-cancer agent [113]. These data were confirmed by another study, which also revealed that DHA treatment in prostate cancer cells down-regulated UHRF1 expression and induced the expression of *p16^INK4A^*, which led to decreased proliferation and metastasis, while enhancing apoptosis [114].

The PI3K/Akt/mTOR (PI3K: phosphoinositide 3-kinase, Akt: serine/threonine kinase 1, and mTOR: mammalian target of rapamycin) signaling pathway has attracted high attention for cancer treatment owing to its role in the differentiation, proliferation, invasion, and migration of cancer cells [115,116,117]. The expression level of mTOR (mammalian target of rapamycin) is high in hepatocellular carcinoma (HCC) and its pharmacological inhibition can reduce tumor cell proliferation [118]. UHRF1 is also overexpressed in HCC and its knockdown leads to the inhibition of migration, proliferation, and epithelial–mesenchymal transition (EMT) in HCC cells [119]. High UHRF1 expression in HCC is linked to poor survival and correlates with immune infiltration [120]. A link between UHRF1 and mTOR was found by Wang et al. [118]. The treatment of HCC cells with the mTOR inhibitor Torin-2 (9-(6-aminopyridin-3-yl)-1-[3-(trifluoromethyl)phenyl]benzo[h][1,6]naphthyridin-2-one) significantly blocked their cellular growth and induced apoptosis, along with the down-regulation of UHRF1. The treatment reduced the expression of UHRF1 at the transcriptional level and thus significantly reduced the UHRF1 protein level. Further studies are needed to explain the exact mechanism involved in Torin-2’s anti-tumor effect. Since mTORC1 (mTOR complex 1) also regulates protein synthesis, it may regulate the translation of unknown factors, which can ultimately affect UHRF1 transcription. Another possibility is that Torin-2 suppresses UHRF1 expression independently of mTORC1 [118]. Torin-2 treatment led to growth inhibition and the induction of apoptosis in colorectal [121], small-cell lung cancer, and non-small-cell lung cancer [122], highlighting the potential of Torin-2 as an anti-cancer agent. Moreover, a recent study reported that UHRF1 silencing could increase the radiosensitivity of ESCC (esophageal squamous cell carcinoma) through the inhibition of the PI3K/Akt/mTOR signaling pathway [123].

### 5.3. Limitations of Synthetic Compounds Targeting UHRF1

There are several considerations before employing these synthetic products for the clinical targeting of UHRF1. These compounds have only been tested *in vitro* or in cell cultures, and more studies are required to validate their efficiency and toxicity *in vivo*. Synthetic molecules that are specifically designed to inhibit UHRF1 activities, drug toxicity, and ease for druggability are important considerations that must be tested in preclinical studies before employing them in clinical settings.

**Table 1:** Synthetic epidrugs targeting UHRF1.

Molecules Directly Targeting UHRF1 Protein				
Drug	Cancers	Mechanism	Function	Reference
**Targeting the SRA domain of UHRF1**				
NSC232003	Glioma	Binds to 5-mC pocket of UHRF1 and inhibits its function	Impaired interaction of UHRF1/DNMT1 Global hypomethylation	[100]
UM63	Cervical	Inhibits recognition and base flipping of 5-mC. Impairs UHRF1/DNMT1 interaction	Reduced global methylation levels	[98]
AMSA2 MPB7	Cervical Melanoma Breast ductal carcinoma	Inhibits UHRF1-SRA-mediated base flipping and recruitment of DNMT1 at replication foci	Reduced global methylation levels and induced apoptosis of cancer cells	[102]
LOPAC compounds (mitoxantrone, doxorubicin, idarubicin, pixantrone, and daunorubicin)	Prostate	Inhibits SRA/HM DNA binding	Global demethylation, synergistic cytotoxic effect on tumor cells in combination with decitabine	[103]
**Targeting the TTD domain of UHRF1**				
BPC		Binds with TTD and favors UHRF1 open conformation	Impaired interaction of UHRF1 with H3K9me3	[104]
NV01 and NV03		Binds with TTD domain of UHRF1	Disrupts UHRF1–H3K9me3 interaction	[105]
5A-DMP	Colorectal	Inhibition of TTD interaction with LIG1	Inhibits interaction of TTD domain with LIG1, which is crucial for maintenance of DNA methylation	[106]
**Synthetic drugs affecting UHRF1 at the transcriptional level and/or protein level**				
LY294002, GF109203X, PD98059, AG490, and genistein. PD0325901 in combination with CHIR99021	Breast, liver, and ALL	Releases the transcription factor E2F to regulate *UHRF1* expression	Inhibition of cellular proliferation and colony formation, cell cycle arrest, and transcriptional regulation of UHRF1 and DNMT1	[109,110]
Dihydroartemisinin	Prostate	Down-regulation of UHRF1 and DNMT1, up-regulation of TSG *p16*	Cell cycle arrest at G1/S, apoptosis, and inhibition of cell proliferation and metastasis	[111,112,113,114]
Torin-2	HCC, colorectal, SCLC, NSCLC, and ESCC	Inhibition of PI3K/Akt/mTOR signaling pathway that regulates UHRF1 expression	Inhibition of migration, proliferation, and EMT, and apoptosis induction	[118,119,120,121,122,123]

**Abbreviations:** UHRF1, Ubiquitin-like, containing PHD and RING finger domains 1; SRA, SET and RING-Associated domain; 5-mC, 5-methylcytosine; DNMT1, DNA-methyltransferase 1; UM63, 2-amino-3-hydroxyanthra-9,10; LOPAC, Library of Pharmacologically Active Compounds; TTD, Tandem Tudor domain; BPC, 4-benzylpiperidine-1-carboximidamide; 5A-DMP, 5-amino-2,4-dimethylpyridine; LIG1, DNA ligase 1; ALL, Acute lymphocytic leukemia; E2F, E2F transcription factor 1; TSG, Tumor-suppressor gene; HCC, Hepatocellular carcinoma; SCLC, Small-cell lung cancer; NSCLC, Non-small-cell lung cancer; ESCC, Esophageal squamous cell carcinoma; EMT, Epithelial–mesenchymal transition.

## 6. Natural Compounds Targeting UHRF1

Since chemotherapy is facing drug-resistance problems and side effects, plant-derived natural compounds can be an interesting alternative in the treatment of cancer. Many studies have shown the potential of natural compounds as anti-cancer agents. For example, taxol, vincristine, camptothecin, and others have proven to be efficient anti-cancer drugs [124,125]. These natural compounds can inhibit tumor cell proliferation and activate apoptosis. There is a long list of natural compounds that either target the regulatory pathways of UHRF1 expression or that directly target UHRF1 (Table 2).

### 6.1. Natural Compounds Directly Targeting UHRF1 Protein

An *in silico* study including molecular dynamics simulation and docking identified two compounds named chicoric acid and nystose with high binding affinities for the SRA domain of UHRF1 [126]. Chicoric acid was found to be a safe and non-toxic compound, that significantly reduced the global methylation levels in colorectal cancer cells [126], but further studies are needed to investigate if it has any potential in treating cancer.

Berberine (BBR) is an alkaloid found in Berberis plants that exhibits anti-tumor activity. A screening approach combining SPR (surface plasmon resonance) and LC-MS/MS (liquid chromatography–tandem mass spectrometry) identified UHRF1 as a potential target of BBR. The alkaloid binds to the TTD and PHD domains of UHRF1 and induces the proteasomal ubiquitin-mediated down-regulation of UHRF1. It also reactivates TSGs, such as *p16^INK4A^* and *TP73*, in multiple-myeloma (MM) cancer cells [127].

A fragment-based ligand discovery study screened an approximately 2300-fragment library to target the TTD. Using binding and functional assays, the study identified 2,4-lutidine as a potential candidate that can bind to two binding pockets located in close proximity to the TTD domain. Through a fragment-linking strategy and medicinal chemistry-based optimization, this compound could be used to develop more potent and specific inhibitors of UHRF1 [128]. Computational studies further reported that taxifolin and luteolin could bind to the SRA domain of UHRF1, disrupting the interaction between SRA and HM DNA [129]. However, since luteolin also down-regulates UHRF1 (see later), it is unlikely that its primary mechanism of action in terms of anti-cancer properties is related to the binding to SRA.

### 6.2. Natural Compounds Affecting UHRF1 Gene Expression

#### 6.2.1. Plant Extracts

*Rhaponticum carthamoides* root extract has shown anti-tumor activity by mediating apoptosis in glioma cells. It induces the cleavage of PARP and inhibits its synthesis, resulting in enhanced DNA damage. Interestingly, the treatment also induces the down-regulation of *UHRF1* and *DNMT1* genes at the mRNA level [130]. These data show the anti-cancer potential of *Rhaponticum carthamoides*, but further studies are needed to establish the molecular mechanisms through which it exerts its anticancer effects, alone or in combination with already known anti-cancer drugs.

The root extract of *Leonurus sibiricus* induces DNA damage, cleavage of PARP, and increased levels of γH2A.X. It also induces the down-regulation of *UHRF1* and *DNMT1* genes in human glioma and U87MG (glioblastoma) cancer cells [131]. This anti-tumoral effect was more significant in the presence of AtPAP1 transcriptional factor derived from *in vitro* transgenic roots transformed by *Agrobacterium rhizogenes*, which may be due to its high content in polyphenolic molecules, such as chlorogenic acid, p-coumaric acid, caffeic acid, and ferulic acid [131]. Further studies on this plant are needed to elucidate the detailed mechanism of its impact on epigenetic regulation and on UHRF1.

Anthocyanins present in *Vaccinium myrtillus* L. (bilberry) possess anti-angiogenesis and anti-tumor activities [132,133]. Antho 50 (bilberry extract enriched with anthocyanins) treatment induces the down-regulation of the UHRF1 protein in B cell chronic lymphocytic leukemic cells and promotes apoptosis through the activation of caspase-3, inhibition of Bcl-2 (B-cell lymphoma), and dephosphorylation of Akt and Bad (BCL2-associated agonist of cell death) [134,135].

The juice of black chokeberry (*Aronia melanocarpa*) has also shown anti-cancer activity in Jurkat cells through a redox-sensitive mechanism. It impedes cellular proliferation and induces cell cycle arrest in the G2/M phase, leading, eventually, to apoptosis. This study noted that the expression of UHRF1 and cyclin B1 decreased, while the expression of p73 and caspase-3 increased after treatment [136].

Maritime pine bark extract (MPTE) contains various polyphenolic components. Our group investigated its anticancer potential and the underlying mechanisms. MPTE shows an inhibitory effect on tumor cell proliferation and induces G2/M phase growth arrest [137]. It plays a pro-oxidant role and induces the expression of p73. Moreover, it down-regulates the expressions of UHRF1 and DNMT1, leading to global DNA hypomethylation. It also promotes dose-dependent apoptosis in cancer cells through the activation of caspase-3, cleavage of PARP, and down-regulation of the anti-apoptotic protein Bcl-2 [137].

Resveratrol, a polyphenol presents in red wine known for its effect against aging, exhibits anti-tumor activity and can induce cell cycle arrest and apoptosis in cancer cells. The treatment of leukemic cells with red wine polyphenols (RWP) decreases cell viability in a concentration-dependent manner and induces cell cycle arrest in the Go/G1 phase [138]. RWP treatment promotes the down-regulation of the UHRF1 protein and activates the expression of p73 and caspase-3 [138]. This treatment increases intracellular ROS (reactive oxygen species) formation, including the formation of superoxide anions, which ultimately regulates the expression of key regulators (p73 and UHRF1) of the cell cycle G1/S transition and apoptosis [138]. Another study reported a chemopreventive effect of grape-derived polyphenols in rats with a C26 colon carcinoma tumor. RWP (red wine polyphenols) treatment down-regulated the expression of UHRF1, while it led to the expression of caspase-3 and TSGs (*p16*, *p53*, and *p73*). Tumor cell proliferation and angiogenesis are inhibited by RWP treatment and, finally, cells undergo apoptosis [133]. Resveratrol, in combination with curcumin, promotes the reactivation of the TSG *PAX1* (Paired box 1) via the down-regulation of UHRF1 [139]. *PAX1* is important for normal chordate development and has been characterized as a tumor-suppressor gene that is frequently hypomethylated in cancers [139]. This study suggests the role of chromatin remodeling in *PAX1* reactivation, which may be mediated through the down-regulation of UHRF1 [139]. Overall, these studies show the anti-cancer potential of red wine polyphenols, either alone or in combination with other anti-cancer drugs.

The aqueous extract of *Limoniastrum guyonianum* shows antioxidant and immunomodulatory activities. This plant contains gallocatechin, epigallocatechin, and epigallocatechin-3-*O*-gallate (EGCG), which exhibit antitumor properties [140]. The treatment of HeLa cells with this extract induces cell cycle arrest at the G2/M phase and apoptosis. The extract effectively down-regulates UHRF1 and DNMT1, resulting in global hypomethylation. It also up-regulates the expression of *p16^INK4A^* [140].

#### 6.2.2. Purified Plant Drugs

Analogues of strigolactone (a plant-derived phytohormone) have been reported to exhibit anti-proliferative and anti-apoptotic activities in HCC [141]. RNA sequencing provided evidence for the effects of the strigolactone analogue named TIT3 on the gene expression of HCC cells. Treatment with TIT3 down-regulated UHRF1 expression in HCC, suggesting that UHRF1 could be a promising target of TIT3. It also down-regulated the expression of DNMT1 and HDAC7 (histone deacetylase 7) and up-regulates several proapoptotic genes [142]. This study also showed that TIT-3 exhibited anti-proliferative and pro-apoptotic activities on HCC cells [142].

Flavonoids are endowed with promising anti-cancer properties. Luteolin (3′,4′,5,7-tetrahydroxyflavone) is a flavonoid compound derived from *Reseda luteola* plants. It has specific anti-proliferative activity and it can lead to cell cycle arrest and apoptosis in cancer cells. Luteolin treatment in colorectal cancer cells not only down-regulates the levels of UHRF1 and DNMT1, but also activates the re-expression of TSG *p16^INK4A^* [140]. Finally, it induces cell cycle arrest and apoptosis by triggering PARP cleavage in tumor cells [140,143]. 

Green tea contains EGCG, which can inhibit UHRF1 and DNMT1 expression [144]. The EGCG-mediated down-regulation of UHRF1 is dependent on ROS generation. The down-regulation of UHRF1 leads to the re-expression of TSGs, such as *p16^INK4A^* and *p73* [144].

A natural naphthoquinone called shikonin is derived from the traditional Chinese herbal medicine *purple gromwell* (Zi cao), known to have anticancer properties. Shikonin promotes cell cycle arrest, apoptosis, and autophagy in different cancer cell lines (A549: lung carcinoma, MDA-MB-231: breast cancer, PANC-1: epithelioid carcinoma, MCF-7: breast adenocarcinoma, HeLa: cervical cancer, and U2OS: osteosarcoma) [145]. In HeLa and MCF-7 cancer cells, shikonin down-regulates UHRF1 expression and up-regulates the expression of *p16^INK4A^* and *TP73*. It prompts apoptosis through p73 and the caspase-3-dependent pathway [146]. Recently, a study described shikonin and melatonin as a promising anti-cancer drug combination [147]. This combination induces oxidative stress-mediated apoptosis in cancer cells through the inhibition of the SIRT3/SOD2-Akt (SIRT3: sirtuin-3, SOD2: superoxide dismutase 2, Akt: serine/threonine kinase 1) pathway [147]. This combination also down-regulates the expression of the UHRF1 protein [147]. However, further studies are needed to investigate the role of UHRF1 in the SIRT3/SOD2-Akt signaling pathway, which regulates various critical cellular processes required for cancer cell survival.

4-Isopropyltropolone (Hinokitiol), found in the essential oil of the *Chymacyparis obtuse* plant, exhibits anti-oxidative, anti-infective, and anti-cancer properties. It inhibits cellular proliferation and induces apoptosis in various tumors [148]. Hinokitiol treatment of colon cancer cells down-regulates UHRF1 and DNMT1 protein expression. It induces the expression of demethylation protein TET1 (ten–eleven translocation methylcytosine dioxygenase 1), which results in the demethylation and activation of various TSGs involved in cell proliferation and biological oxidation [148].

Emodin (C15H10O5) is an active constituent obtained from the rhizomes of Chinese herbs used in various diseases, including cancer. It induces growth arrest and apoptosis in tumor cells. On lymphoma Raji cells, emodin decreases the cell viability and UHRF1 protein levels. It induces apoptosis through the activation of caspase-3, caspase-9, and PARP1. Emodin decreases *p73* promoter 2 activity through the inhibition of UHRF1 and suppresses the activity of p73-Luc-2 by up-regulating DNMT3A (DNA methyltransferase 3A) [149]. Emodin in combination with doxorubicin induces apoptosis, indicating that emodin can sensitize tumor cells to doxorubicin treatment [149].

Recently, a screening study identified a natural compound from traditional Chinese medicine named diosgenin (DSG) as an inhibitor of UHRF1 [150]. DSG interacts with UHRF1 and impairs its interaction with USP7 [150]. As a result, UHRF1 is degraded through the ubiquitin–proteasome pathway. DSG down-regulates the DNA methylation levels and activates the TSGs *p21*, *p16*, and *LXN* (latexin). The treatment of prostate cancer cells with DSG leads to cell cycle arrest, senescence and inhibition of cellular proliferation, and xenograft tumor growth [150]. Further development of DSG derivatives with improved affinity for UHRF1 and inhibitory action on UHRF1 activity could lead to a promising strategy against cancer.

### 6.3. Natural Compounds Targeting UHRF1 at Gene and Protein Levels

Thymoquinone (TQ), a bioactive constituent of the volatile oil of *Nigella sativa* (black seeds), shows anti-cancer activity against different types of cancers [151,152,153]. It inhibits cell growth and favors cell cycle arrest, leading to p53-dependent and p53-independent apoptosis in tumor cells. It also promotes the re-expression of the TSG *p16* [154,155,156]. It also induces apoptosis through the activation of *TP73* [157]. TQ also induces the ubiquitination-mediated degradation of UHRF1, with a parallel decrease in USP7 (de-ubiquitinase enzyme) expression. UHRF1 down-regulation is also accompanied by an increase in p73 and caspase-3 expression [158]. Interestingly, TQ down-regulates different components of the macromolecular complex “ECREM” (Epigenetic Code Replication Machinery) [88]. Using molecular docking, Polepalli et al. [159] suggested that TQ likely binds within the 5-mC binding pocket of the SRA domain of UHRF1 [159].

Naphthazarin (5,8-dihydroxy-1,4-naphthoquinone, DHNQ, Naph) is a 1,4-naphthoquinone derivative of plant origin having antioxidant, anti-inflammatory, and anti-cancer properties [160]. Naphthazarin decreases the cell viability of human MCF-7 tumor cells [160]. In combination with ionizing radiation, naphthazarin increases p53-dependent p21 expression and down-regulates UHRF1, DNMT1, and HDAC1 (histone deacetylase 1) at both the mRNA and protein levels. Naphthazarin appears as a promising radiosensitizer in breast cancer [160]. A recent study reported that UHRF1 modulates the growth of breast tumor cells through estrogen signaling, and its depletion significantly inhibits breast cancer cell growth *in vitro* and *in vivo*. Clinical data have shown that UHRF1 is overexpressed and correlates with poor survival in luminal-type breast cancer patients [161]. UHRF1 interacts with ERα (estrogen receptor alpha) through its SRA domain and stabilizes ERα by inhibiting its ubiquitination [161]. This suggests a novel role of UHRF1 in luminal-type breast cancer, but further studies are needed to investigate the effect of naphthazarin or its derivatives against this cancer. Moreover, it has been reported that naphthazarin in combination with desatinib, an inhibitor of the pre-B cell receptor, regulates the expression of UHRF1 and ROR1 (transmembrane pseudokinase) in lymphoblastic leukemia cancer, leading to significantly reduced cell viability [162]. The expression of ROR1 is crucial for the survival of acute lymphoblastic leukemia, chronic lymphocytic leukemia, and various solid cancers. Targeting ROR1 with small molecules is challenging because of the absence of ROR1 kinase activity [162]. UHRF1 regulates and maintains the levels of ROR1 and is required for the viability of lymphoblastic leukemia through a mechanism associated with ROR1 expression. The inhibition of UHRF1 in combination with desatinib leads to a decrease in the ROR1 levels, suggesting a novel mechanism to target acute lymphoblastic leukemia [162].

Plumbagin is a naphthoquinone derived from the *Plumbago zeylanica* plant that exhibits anticancer properties. Plumbagin down-regulates the expression of UHRF1 at both the transcript and protein levels, which leads to the inhibition of metastasis and inhibition of the proliferation of cervical cancer cells [163]. It down-regulates *E2F1* and up-regulates *p21*, leading to cell cycle arrest at the G2/M phase. Plumbagin down-regulates the expression of *Akt-1*, *PARP-1,* and *MMP-2* (metalloproteinase 2) and up-regulates the expression of *caspase 9* and TIMP-2 (tissue inhibitor of MMP-2), ultimately inducing apoptosis in tumor cells. Plumbagin in combination with cisplatin shows a synergistic effect promoting apoptosis [163]. However, *in vivo* studies are needed to confirm the anti-cancer activity of plumbagin against cervical cancer.

Curcumin isolated from *Curcuma longa* (turmeric) has shown anti-tumor activity in both *in vitro* and *in vivo* models [164]. The target of curcumin is phosphodiesterase 1A (PDE1A), which plays an important role in the proliferation of melanoma cells. Curcumin down-regulates PDE1A, as well as UHRF1, DNMT1, and cyclin A. Curcumin blocks the cell cycle and reactivates TSGs (p21 and p27), presumably due to the down-regulation of UHRF1 resulting from PDE1A repression [164]. Interestingly, the overexpression of PDE1A up-regulates the expression of UHRF1 and DNMT1, and also inhibits the antiproliferative effect of curcumin in B16F10 (murine melanoma) cells [164]. These data highlight the novel molecular mechanism through which curcumin can regulate the epigenetic control of gene expression and inhibit cancer [164]. Curcuminoids (CCMs, polyphenols in curcuma), in combination with sodium butyrate, a histone deacetylase inhibitor naturally present in the human body, synergistically decrease the viability of glioblastoma cells [165]. Indeed, they induce apoptosis, cell cycle arrest, and ROS generation in parallel with the down-regulation of UHRF1 gene expression [165]. These studies reveal promising anti-tumoral activity of curcumin and its potential to prevent cancer occurrence through the epigenetic control of gene expression.

#### Natural Compounds Purified from Bacteria

Anisomycin, an antibiotic isolated from *Streptomyces*, inhibits the growth of Jurkat cancer cells. It down-regulates UHRF1 and induces cell cycle arrest at the S and G2/M phases through the inhibition of P-CDK2 (phospho-cyclin-dependent kinase 2) in a dose-dependent manner. It also reactivates the expression of TSGs: p21, p27, and p53 [166].

The *UHRF1* gene is also overexpressed in malignant pleural mesotheliomas (MPM) and is correlated with poor survival in MPM patients. In contrast, *UHRF1* knockdown inhibits the proliferation, invasion, and growth of tumor xenografts and reverses global DNA hypomethylation. *UHRF1* expression is significantly inhibited by treatment with repurposed chemotherapeutic drugs, such as mithramycin (a DNA-binding antitumor antibiotic produced by the bacterium *Streptomyces plicatus*), in MPM cells, suggesting *UHRF1* as a druggable target in mesotheliomas [167]. However, further studies are needed to assess the role of *UHRF1* in the pathogenesis and prognosis of MPM, as well as to investigate the potential of mithramycin to target *UHRF1* alone or in combination with inhibitors of its epigenetic partners.

### 6.4. Limitations of Natural Compounds Targeting UHRF1

Natural compounds derived from plants have been studied for their potential anti-tumoral properties for a few decades. Before using the natural compounds for the clinical targeting of UHRF1, we should keep in mind some limitations associated with these compounds. Natural compounds can serve as lead molecules to downregulate UHRF1, but, at the same time, they can also target multiple genes and pathways, which indicates the complexity of biological effects. It is important to screen all the extracts’ constituents to identify the specific compounds that are involved in the regulation of UHRF1. Similarly, many of the natural compounds listed in the table have already been reported for various pharmacological activities, so there is a need to identify the precise mechanism through which these compounds exert their anti-UHRF1 effect. There is also a need to improve the solubility, metabolism rate, and bioavailability of natural compounds and to opt for advanced drug delivery systems to deliver the drug in a precise manner.

**Table 2:** Natural epidrugs targeting UHRF1 expression.

Natural Compounds Directly Targeting UHRF1 Protein				
Drug	Cancers	Mechanism	Function	Reference
Chicoric acid	Colorectal	Binds with SRA domain and inhibits its activity	Reduces methylation levels	[126]
Berberine	Multiple myeloma	Binds with TTD and PHD domain of UHRF1 and induces ubiquitination-mediated degradation of UHRF1, activates *p16^INK4A^* and *p73*	Inhibits cell growth and cytotoxic in multiple myeloma cells	[127]
2,4-Lutidine		Inhibits binding of TTD with H3K9me3	Inhibits H3K9me3 mark recognition by TTD and may induce the expression of TSGs	[128]
**Natural Compounds Affecting *UHRF1* Gene Expression**				
**Plant extracts**				
*Rhaponticum carthamoides* root extract	Glioma	Down-regulation of *UHRF1* and *DNMT1* mRNA levels, cleavage of PARP, and inhibition of PARP synthesis	Apoptosis	[130]
*Leonurus sibiricus* root extract	Glioma	In combination with AtPAP1, transcription factor induces down-regulation of *UHRF1* and *DNMT1*, cleavage of PARP, and an increase in γH2A.X.	DNA damage	[131]
*Vaccinium myrtillus*, Bilberry extract, *Aronia melanocarpa*	B cell chronic lymphocytic leukemia and Jurkat cells	Down-regulation of UHRF1 and Cyclin B1, activation of p73 and caspase-3 expression, inhibition of Bcl-2, and dephosphorylation of Akt and Bad	Anti-tumor, anti-angiogenesis, anti-proliferative, and cell cycle arrest in G2/M phase	[132,134,135,136]
Maritime pine tannin extract	Cervical and osteosarcoma	Down-regulation of UHRF1 and DNMT1, induces expression of p73 and caspase-3, cleavage of PARP, and down-regulation of pro-apoptotic Bcl-2	Anti-proliferative, G2/M phase growth arrest, global hypomethylation, and apoptosis	[137]
Red wine-derived polyphenols	Leukemia and C26 carcinoma	Down-regulation of UHRF1 and induces expression of TSGs: *p16*, *p53*, *p73*, *PAX1*, and caspase-3 protein	Reduced cell viability, cell cycle arrest, apoptosis, inhibition of proliferation, and angiogenesis	[133,138,139]
*Limoniastrum guyonianum*	Cervical	Down-regulates UHRF1 and DNMT1, activates the expression of TSG *p16^INK4A^*	Cell cycle arrest at G2/M phase, apoptosis, and global hypomethylation	[140]
**Purified plant drugs**				
TIT3	HCC	Down-regulation of *UHRF1*, *DNMT1*, *HDAC7,* and DNA repair genes, and up-regulation of proapoptotic genes	Anti-proliferative and proapoptotic effect	[142]
Luteolin	Colorectal	Down-regulation of UHRF1 and DNMT1, re-expression of TSG *p16^INK4A^*, and PARP cleavage.	Antiproliferative, cell cycle arrest, and apoptosis	[140,143]
EGCG	ALL	ROS-dependent down-regulation of UHRF1 and DNMT1, and re-expression of TSGs: p73 and *p16^INK4A^*	Cell cycle arrest and apoptosis	[144]
Shikonin (alone or in combination with melatonin)	NSCLC, breast, pancreatic, cervical, and osteosarcoma	Down-regulation of UHRF1, re-expression of TSG *p16^INK4A^*, p73 and caspase-3-dependent apoptosis, and oxidative stress mediated-apoptosis	Cell cycle arrest, apoptosis, and autophagy	[145,146,147]
Hinokitiol	Colon	Down-regulation of UHRF1 and DNMT1, and induces expression of TET1 protein and TSGs involved in cell proliferation	Anti-proliferative, apoptosis, and demethylation	[148]
Emodin (alone or in combination with doxorubicin)	Lymphoma Raji cells	Inhibition of UHRF1 expression and activation of caspase-3, caspase-9, and PARP	Growth arrest, apoptosis, reduced cell viability, and enhanced tumor cell sensitivity to doxorubicin	[149]
Diosgenin	Prostate	Ubiquitin-mediated degradation of UHRF1, down-regulation of DNA methylation, and activation of TSG: *p16*, *p21,* and *LXN*	Cell cycle arrest, senescence, and inhibition of cellular proliferation and xenograft tumor growth	[150]
**Natural Compounds Targeting UHRF1 at Gene and Protein Level**				
Thymoquinone	Renal, colorectal, osteosarcoma, astrocytoma, ovarian adenocarcinoma, cervical, and breast	Down-regulation of UHRF1/DNMT1/HDAC1/G9a/USP7 and re-expression of TSGs: *p16* and *p73*. Induces expression of caspase-3	Anti-proliferative, cell cycle arrest, and p53-dependent and p53-independent apoptosis	[151,152,153,154,155,156,157,158,159]
Naphthazarin	Breast and t(1;19)-pre-B-cell acute lymphoblastic leukemia	Down-regulation of UHRF1/DNMT1/HDAC1, induces p53-dependent p21 expression, and inhibition of ROR1 expression	Cell cycle arrest, apoptosis, enhances radio sensitivity of MCF-7 breast cancer cells, and reduces ALL cell viability	[160,162]
Plumbagin (alone or in combination with cisplatin)	Cervical	Down-regulation of UHRF1 at transcript and protein levels and down-regulation of *Akt-1*, *caspase 9,* and *PARP1*	Inhibition of metastasis and proliferation, and synergistic apoptosis in combination with cisplatin	[163]
Curcumin	Melanoma and glioblastoma	Targets PDE1 enzyme, down-regulation of UHRF1, DNMT1, and cyclin A, activation of *p21*, *p27,* and *PAX1*, and ROS generation	Anti-proliferative, cell cycle arrest, reduced viability of glioblastoma cells in combination with sodium butyrate, and apoptosis	[139,164,165]
**Natural compounds purified from bacteria**				
Anisomycin	ALL	Down-regulation of UHRF1 and activation of TSGs: *p21*, *p27*, and *p53*	Cell cycle arrest at G2/M phase	[166]
Mithramycin	Malignant pleural mesotheliomas	Down-regulation of *UHRF1*	Targeting DNA methylation	[167]

**Abbreviations:** UHRF1, Ubiquitin-like, containing PHD and RING finger domains 1; SRA, SET and RING-associated domain; TTD, Tandem Tudor domain; PHD, Plant homeodomain; TSGs, Tumor-suppressor genes; DNMT1, DNA-methyltransferase 1; PARP, Poly (ADP-ribose) polymerase; AtPAP1, Transcription factor from Arabidopsis thaliana; Bcl-2, B-cell lymphoma 2; Bad, BCL2-associated agonist of cell death; PAX1, Paired box protein Pax-1; HCC, Hepatocellular carcinoma; EGCG, Epigallocatechin-3-O-gallate; ALL, Acute lymphocytic leukemia; ROS, Reactive oxygen species; NSCLC, Non-small-cell lung cancer; TET1, Ten–eleven translocation methylcytosine dioxygenase 1; LXN, Latexin; HDAC1, Histone deactylase 1; USP7, Ubiquitin-specific-processing protease 7; ROR1, transmembrane pseudokinase; MCF-7, Michigan Cancer Foundation-7; PDE1, Phosphodiesterase 1.

## 7. Conclusions and Prospects

Recent breakthroughs in epigenetics have shed light on the importance of epigenetic regulation in various human diseases, with a special emphasis on cancer. The underlying mechanisms of epigenetic regulation have gained attention as a potential therapeutic strategy to target various types of cancers. Dysregulations in the DNA methylome can promote the oncogenic transformation of cells in the body [4,5,6,7]. Therefore, a detailed understanding of epigenetic regulations and the identification of the key players involved can help to design new therapeutic drugs and/or cancer biomarkers. This present review provides insight into the molecular mechanisms underlying how natural and synthetic molecules can be sources of novel therapies by inhibiting UHRF1 expression and/or activity. The development of small-molecule inhibitors that can bind to the different domains of UHRF1 is promising, as UHRF1 is a master regulator of the epigenetic code transmission in coordination with several epigenetic proteins. Of note, it is highly interesting to observe that, when a drug directly targets UHRF1, it often leads to reduced levels of this protein. This warrants further investigation, as there might be a possibility that the direct binding of drug molecules with UHRF1 hinders its interaction with protective proteins, such as deubiquitylases, e.g., USP7, thus reducing the levels of this protein.

The epigenetic integrator UHRF1 is overexpressed in nearly all cancer types, gaining the status of a potential oncogenic protein and being likely to become a universal biomarker for cancer [15,77]. The aberrant expression of UHRF1 breaks the “epigenetic code”, leading to the silencing of tumor-suppressor genes (TSGs) through promoter hypermethylation, accumulation, or repressive histone marks. As a consequence, UHRF1 overexpression is involved in tumor proliferation, the inhibition of DNA repair, and the development of resistance toward anti-cancer therapy. Therefore, UHRF1 can be a pragmatic/attractive anti-cancer target [12,14,77,168,169]. Accordingly, we believe that targeting UHRF1 is interesting because the latter is well-known to silence a huge number of TSGs in a non-irreversible fashion. Indeed, the down-regulation of UHRF1 always allows the recovery of almost all TSGs’ expressions [77]. This is particularly relevant for the future discovery of anti-cancer drugs, because we think that favoring cancer cells to commit suicide is better than the current chemotherapeutic approach to cancer treatment. Indeed, the latter poses a multitude of challenges due to the low selectivity and genotoxicity often leading to treatment-induced tumor formation and to severe side effects [170]. Therefore, we further think that the re-expression of TSGs through the down-regulation of UHRF1 by the activation of the apoptotic pathway will be more bearable by patients, as much fewer side effects will be expected. Nevertheless, the efficiency of the drug might depend on the level of UHRF1 found in each type of cancer [77].

A large number of natural drugs antagonize the UHRF1 effects *in vitro*, but need to be improved in order to be employed for anti-cancer therapy. Indeed, there are several drawbacks of natural compounds, e.g., curcumin and flavonoids, such as quick systemic elimination, poor pharmacokinetics, poor absorption, low water solubility, fast metabolism, low bioavailability, low penetration-targeting efficacy, and instability against environmental factors [171,172]. The use of nano-carriers can resolve these issues [171], as it has been also suggested for curcumin and flavonoids [171,173].

We propose that natural compounds targeting UHRF1 (as a direct inhibitor or regulating its expression or stability) may serve as lead compounds for designing highly active drugs through chemical modifications. This would help to design new highly selective anti-cancer drugs, but their high selectivity leads to significant drawbacks due to the intrinsic tumor heterogeneity. It has been reported that plant polyphenols can simultaneously affect many processes that are involved in acquiring and maintaining the hallmark properties of malignant cells, and their toxic dose is typically much higher than the therapeutic one [170]. To overcome these issues, we propose that the use of a combination of several natural drugs targeting UHRF1 will be an interesting future direction to take, considering that synergistic effects can be expected. Indeed, mixtures of natural compounds are often more active than single molecules because they contain several active compounds that may synergize to exert their effects [170]. Despite being an attractive target, the development of novel molecules that can precisely target UHRF1 and can reach clinical trials is not easy. The biggest challenge for drugs targeting epigenetic machinery is the development of adverse reactions, either because the target is not specifically expressed in tumors, or the drug is unable to inhibit the activity of the target protein specifically. In the case of UHRF1, the problem of specificity can be addressed efficiently, as UHRF1 expression is up-regulated in a variety of cancers in comparison with normal cells. The remaining concern is to identify and develop molecules that can specifically target UHRF1. The development of reliable and efficient models can be very helpful to test and predict novel molecules alone or in combination with other drugs to target UHRF1, accelerating the entry of promising molecules in clinical trials.

Similarly, advanced drug screening and design (including virtual library and high-throughput screening, bioinformatics, and structure-based tools) are necessary to develop more specific and direct inhibitors of UHRF1 with a broad spectrum of activity against various cancers with minimal off-target effects. In addition, further investigations are also needed to fully assess the exact mechanism by which these natural drugs or synthetic compounds reverse the effect of UHRF1 overexpression in cancer. They might notably either induce the degradation of UHRF1 or re-expression of tumor suppressor genes, or hinder genomic repair in cancerous cells induced by cytotoxic drugs. Natural and synthetic inhibitors and their solved structures with UHRF1 domains can serve as a starting point for future screening and hit-to-lead optimization. This will open new horizons in cancer research by targeting cellular epigenetic abnormalities that contribute to uncontrolled proliferation and oncogenesis.

## Data Availability

This study did not report any data.
